# Comparative genomics of bacterial and plant folate synthesis and salvage: predictions and validations

**DOI:** 10.1186/1471-2164-8-245

**Published:** 2007-07-23

**Authors:** Valérie de Crécy-Lagard, Basma El Yacoubi, Rocío Díaz de la Garza, Alexandre Noiriel, Andrew D Hanson

**Affiliations:** 1 Department of Microbiology and Cell Science, University of Florida, Gainesville, FL 32611, USA; 2 Department of Horticultural Sciences, University of Florida, Gainesville, FL 32611, USA

## Abstract

**Background:**

Folate synthesis and salvage pathways are relatively well known from classical biochemistry and genetics but they have not been subjected to comparative genomic analysis. The availability of genome sequences from hundreds of diverse bacteria, and from *Arabidopsis thaliana*, enabled such an analysis using the SEED database and its tools. This study reports the results of the analysis and integrates them with new and existing experimental data.

**Results:**

Based on sequence similarity and the clustering, fusion, and phylogenetic distribution of genes, several functional predictions emerged from this analysis. For bacteria, these included the existence of novel GTP cyclohydrolase I and folylpolyglutamate synthase gene families, and of a trifunctional *p*-aminobenzoate synthesis gene. For plants and bacteria, the predictions comprised the identities of a 'missing' folate synthesis gene (*folQ*) and of a folate transporter, and the absence from plants of a folate salvage enzyme. Genetic and biochemical tests bore out these predictions.

**Conclusion:**

For bacteria, these results demonstrate that much can be learnt from comparative genomics, even for well-explored primary metabolic pathways. For plants, the findings particularly illustrate the potential for rapid functional assignment of unknown genes that have prokaryotic homologs, by analyzing which genes are associated with the latter. More generally, our data indicate how combined genomic analysis of both plants and prokaryotes can be more powerful than isolated examination of either group alone.

## Background

**Figure 1:**
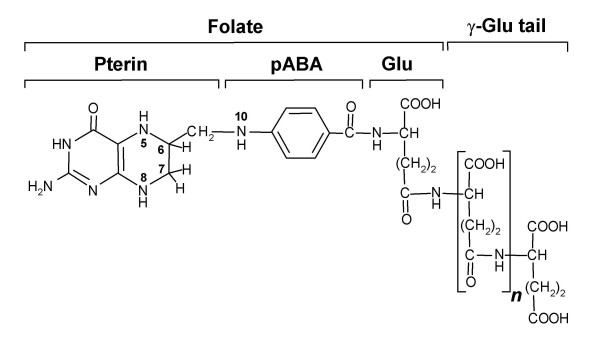
**The structure of tetrahydrofolate**. In natural folates, the pterin ring exists in tetrahydro form (as shown) or in 7,8-dihydro form (as in DHF). The ring is fully oxidized in folic acid, which is not a natural folate. Folates usually have a γ-linked polyglutamyl tail of up to about eight residues attached to the first glutamate. One-carbon units (formyl, methyl, etc.) can be coupled to the N5 and/or N10 positions.

Folates are tripartite molecules comprising pterin, *p*-aminobenzoate (pABA), and glutamate moieties to which one-carbon units at various oxidation levels can be attached at the N5 and N10 positions (Figure 1). In natural folates the pterin ring is in the dihydro or tetrahydro state, and a short, γ-linked polyglutamyl tail of up to about eight residues is usually attached to the first glutamate.

**Figure 2:**
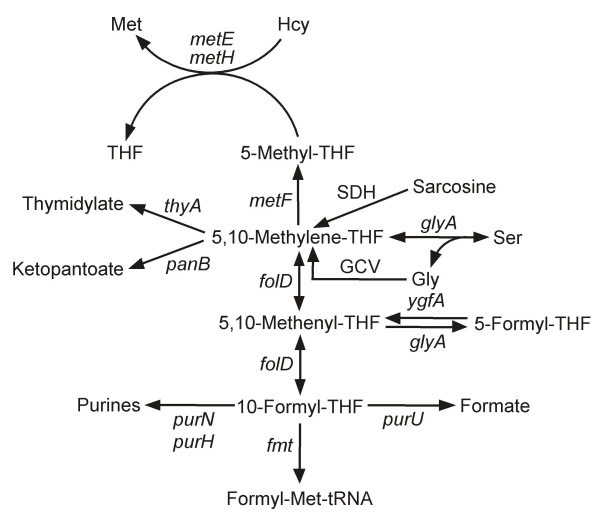
**Major folate-dependent reactions of one-carbon metabolism**. The gene names are for *E. coli *(except for sarcosine dehydrogenase). Note that the formation of 5-formyl-THF from 5,10-methenyl-THF occurs via a second catalytic activity of serine hydroxymethyltransferase (*glyA*), and that 5-formyl-THF is reconverted to 5,10-methenyl-THF by 5-formyl-THF cycloligase (*ygfA*). For simplicity, THF is not shown as a participant in most reactions in which it is consumed or released. GCV, glycine cleavage complex, comprising the products of the *gcvT*, *gcvH*, *gcvP*, and *lpd *genes; SDH, sarcosine dehydrogenase (not present in *E. coli*).

Tetrahydrofolates serve as cofactors in one-carbon transfer reactions during the synthesis of purines, formylmethionyl-tRNA, thymidylate, pantothenate, glycine, serine, and methionine [1] (Figure 2). Most folate-dependent enzymes strongly prefer polyglutamates to monoglutamates, but the opposite is usually true of folate transporters so that polyglutamylation is generally considered to favor folate retention within cells and subcellular compartments [2,3].

Plants, fungi, certain protists, and most bacteria make folates *de novo*, starting from GTP and chorismate, but higher animals lack key enzymes of the synthetic pathway and so require dietary folate [4-7]. Folates are crucial to human nutrition and health [3], and antifolate drugs are widely used in cancer chemotherapy and as antimicrobials [3,7,8]. For these reasons, folate synthesis and salvage pathways have been extensively characterized in model organisms, and the folate synthesis pathway in both bacteria and plants has been engineered in order to boost the folate content of foods [9-11].

**Figure 3:**
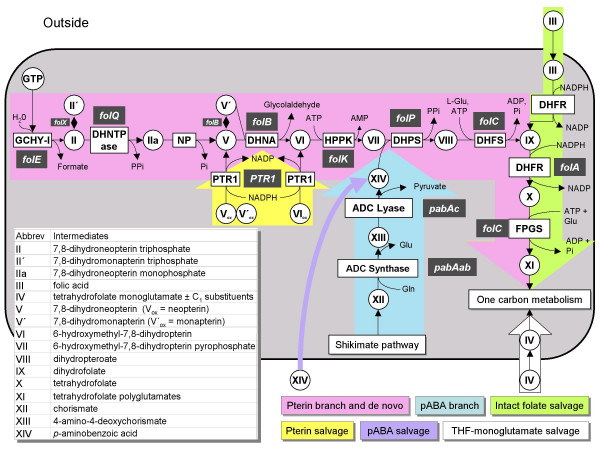
**Folate synthesis and salvage pathways**. Gene names are white-on-gray; all except *folQ *and *PTR1 *are from *E. coli*. The *folQ *gene has been identified only in *Lactococcus lactis *and plants, and *PTR1 *only in *Leishmania *and other trypanosomatids. Note that DHN aldolase also mediates epimerization of DHN to 7,8-dihydromonapterin and aldol cleavage of 7,8-dihydromonapterin. ADC, aminodeoxychorismate; DHFR, dihydrofolate reductase; DHFS, dihydrofolate synthase; DHNA, dihydroneopterin aldolase; DHNTPase, dihydroneopterin triphosphate pyrophosphatase; DHPS, dihydropteroate synthase; FPGS, folylpolyglutamyl synthase; HPPK, hydroxymethyldihydropterin pyrophosphokinase; NP, nonspecific phosphatase; PTR1, pteridine reductase 1; the subscript _ox _denotes the fully oxidized forms of pterins.

The *de novo *folate synthesis pathway has the same steps in bacteria and plants, and consists of a pterin branch and a pABA branch (Figure 3, rose and blue color, respectively). The first enzyme of the pterin branch is GTP cyclohydrolase I (GCHY-I, EC 3.5.4.16), which catalyzes a complex reaction in which the five-membered imidazole ring of GTP is opened, C8 is expelled as formate, and a six-membered dihydropyrazine ring is formed using C1 and C2 of the ribose moiety of GTP [5]. The resulting 7,8-dihydroneopterin triphosphate is then converted to the corresponding monophosphate by a specific pyrophosphatase [5,12]. Removal of the last phosphate is believed to be mediated by a non-specific phosphatase [5]. Dihydroneopterin aldolase (DHNA, EC 4.1.2.25) then releases glycolaldehyde to produce 6-hydroxymethyl-7,8-dihydropterin, which is then pyrophosphorylated by hydroxymethyldihydropterin pyrophosphokinase (HPPK, EC 2.7.6.3). DHNA also interconverts 7,8-dihydroneopterin and 7,8-dihydromonapterin, and cleaves the latter to 6-hydroxymethyl-7,8-dihydropterin. A paralog of DHNA, FolX, interconverts the triphosphates of 7,8-dihydroneopterin and 7,8-dihydromonapterin, and also catalyzes the same reactions as DHNA at very slow rates [13].

In the pABA branch of the pathway, chorismate is aminated to aminodeoxychorismate (ADC) by ADC synthase (EC 6.3.5.8) using the amide group of glutamine as amino donor [5]. ADC is then converted to pABA by ADC lyase (EC 4.1.3.38) [5].

6-Hydroxymethyl-7,8-dihydropterin pyrophosphate and pABA moieties are condensed by dihydropteroate synthase (DHPS, EC 2.5.1.15). The resulting dihydropteroate is glutamylated by dihydrofolate synthase (DHFS, EC 6.3.2.12) giving dihydrofolate (DHF), which is reduced by dihydrofolate reductase (DHFR, EC 1.5.1.3) to tetrahydrofolate (THF). Folylpolyglutamate synthase (FPGS, EC 6.3.2.17) then adds a γ-glutamyl tail. In *Escherichia coli*, it has been reported that there can also be α linkages in the distal part of the polyglutamyl tail [14].

**Figure 4:**
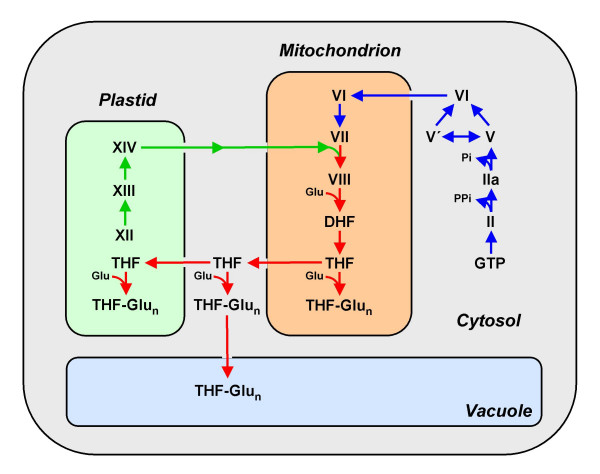
**Compartmentation of the folate synthesis pathway in plants**. The steps in the pterin branch of the pathway are in blue, those in the pABA branch are in green, and the others (condensation, glutamylation, and reduction) are in red. Note that the compartmentation of the pathway and of its folate end products implies the existence of pterin or folate carriers in the mitochondria, chloroplast, and vacuolar membranes. Pathway intermediates are designated by the symbols used in Figure 3. THF-Glu_n_, THF polyglutamates.

Although the biosynthetic steps are the same in plants and bacteria, the plant pathway is split between three subcellular compartments, with pterin synthesis in the cytosol, pABA synthesis in chloroplasts, and the other steps in mitochondria (Figure 4) [6]. FPGS isoforms are present in all three of these compartments, as are folates themselves [15,16]. Folates – both poly- and monoglutamates – are also found in plant vacuoles [16]. The highly compartmented nature of folate synthesis in plants implies the existence of pterin and folate transporters that are integral components of the pathway.

Folate-related salvage pathways are of three kinds. The first ('intact folate salvage') (Figure 3, green color) enables utilization of supplied folic acid and DHF, and relies on a DHFR activity to reduce these oxidized folates to THF, and on an FPGS activity [7]. DHFR activity is also required to recycle the DHF produced in the reaction catalyzed by thymidylate synthase (TS, EC 2.1.1.45). The second kind of salvage ('pterin salvage') (Figure 3, yellow color), known in *Leishmania *and other trypanosomatid parasites, involves the reduction of fully oxidized pterins to the dihydro and tetrahydro levels by pteridine reductase 1 (PTR1, EC 1.5.1.33) [17]. This enables oxidized pterins to be used (after reduction to dihydro forms) for folate synthesis, and (after reduction to tetrahydro forms) as cofactors for aromatic hydroxylases and other pterin-dependent enzymes. Finally, some bacteria, plants, and protists probably carry out a more radical kind of salvage, in which the pterin and pABA-glutamate fragments produced by folate breakdown are recycled for folate synthesis [18]. This type of salvage has been little studied and will not be considered further in this article.

Genes for all the enzymes of folate synthesis have been identified in model organisms such as *Escherichia coli, Saccharomyces cerevisiae*, and *Arabidopsis thaliana *[4-6]. Likewise, the intact folate salvage pathway has been well characterized in mammals, the malaria parasite *Plasmodium*, and *Lactobacillus casei *[7,19,20], and pterin salvage in *Leishmania *[17]. However, analysis of the distribution of known folate synthesis and salvage genes in hundreds of bacterial genomes using the SEED platform [21] reveals that much remains to be learnt about both synthesis and salvage.

The SEED is a freely available, open-source database that provides efficient ways to discover new genes or pathways, to generate predictions about gene function, and to improve annotations, based on a 'functional subsystem approach' [21]. This approach has much in common with metabolic reconstruction [22,23]. A *functional subsystem *may be defined as a set of functional roles (usually ten to twenty) jointly involved in a biological process. A typical subsystem is a group of enzymes, transporters, and regulatory components that participate in a metabolic pathway such as folate synthesis or salvage. Subsystem analysis examines which components are actually present in a genome and which should be present but cannot be identified, and so provides a picture of what is actually *missing*. This sets the stage to pursue the 'missing genes', also termed 'pathway holes' [24-26]. Homology-based searches alone are usually unable to locate missing genes that have not been previously identified in any genome ('globally missing genes') or those that are missing due to non-orthologous gene replacement ('locally missing genes') [27].

In this study, we first predicted the pathways (*de novo *folate synthesis, intact folate salvage, and pterin salvage) present in around four hundred sequenced bacteria and identified cases of missing genes for almost every step of the synthesis pathway. Candidates for such missing genes in bacteria and plants were then predicted using comparative genomic tools and representative candidates were tested experimentally.

## Results and Discussion

### Are folates essential in all bacteria?

As folate-dependent formylation of the initiator tRNA is a hallmark of bacterial translation and bacteria cannot import formylmethionyl-tRNA [28], we investigated the distribution of the *fmt *gene encoding methionyl-tRNA formyltransferase (EC 2.1.2.9) as a signature gene for a folate requirement. Homologs of *fmt *are found in all sequenced genomes except *Mycoplasma hyopneumoniae *and Onion yellows phytoplasma OY-M (Table 1). We confirmed the observation [29] that *M. hyopneumoniae *lacks all the enzymes of folate-mediated one-carbon metabolism except for glycine hydroxymethyltransferase (GlyA), which has aldolase activities that do not require folate [30]. Another widespread folate-dependent metabolic step is the conversion of dTMP to dUMP, catalyzed by thymidylate synthase (ThyA, EC 2.1.1.45). This step can also be performed by a folate- and flavin-dependent thymidylate synthase (ThyX) [31]. As first observed by Myllykallio et al. [32], most bacteria have a *thyA *or a *thyX *homolog, some have both, and the few that have neither – such as *M. hyopneumoniae *or *Ureaplasma parvum *– contain the *tdk *gene encoding the thymidine (dT) salvage enzyme thymidine kinase. Our genomic analysis suggests that *M. hyopneumoniae *strains are the only sequenced bacteria that do not require folate for initiator tRNA formylation or thymidylate synthesis. The situation in the phytoplasma that lacks the *fmt *gene (Table 1) is different; it contains a *thyA *homolog like most *Mycoplasma *species and therefore presumably requires intact folates.

**Table 1:** Examples of bacteria dependent on folate salvage

***Organism***	***De novo signature enzymes***		***Folate salvage pathway***		***Folate requiring enzymes***			***Folate independent dTMP synthesis***	
	**FolK**	**FolP**	**Dhfr^a^**	**FolC^b^**	**ThyA**	**GlyA**	**Fmt**	**ThyX**	**Tdk**
	
*Lactobacillus acidophilus*	**-**	**-**	**+**	**+**	**+**	**+**	**+**	**-**	**+**
*Pediococcus pentosaceus*	**-**	**-**	**+**	**+**	**+**	**+**	**+**	**-**	**+**
*Buchnera aphidicola *(3 strains)	**-**	**-**	**+**	**+**	**+**	**+**	**+**	**-**	**-**
*Rickettsia rickettsii*	**-**	**-**	**?**	**+**	**-**	**+**	**+**	**+**	**-**
*Bartonella henselae *str. Houston-1	**-**	**-**	**-**	**-**	**+**	**+**	**+**	**-**	**-**
*Mycoplasma hyopneumoniae *(3 strains)	**-**	**-**	**-**	**-**	**-**	**+**	**-**	**-**	**+**
*Mycoplasma genitalium *G-37	**-**	**-**	**+**	**-**	**+**	**+**	**+**	**-**	**+**
*Mycoplasma synoviae *53	**-**	**-**	**+**	**-**	**+**	**-**	**+**	**-**	**+**
*Ureaplasma parvum *ATCC 700970	**-**	**-**	**+**	**-**	**-**	**-**	**+**	**-**	**+**
*Mesoplasma florum *L1	**-**	**-**	**+**	**+**	**+**	**+**	**+**	**-**	**+**
*Onion yellows phytoplasma *OY-M	**-**	**-**	**+**	**+**	**+**	**+**	**-**	**-**	**+**
*Spiroplasma kunkelii *CR2-3x	**-**	**-**	**+**	**-**	**+**	**+**	**+**	**-**	**+**
*Borrelia burgdorferi *B31	**-**	**-**	**?**	**-**	**-**	**+**	**+**	**+**	**-**
*Treponema pallidum *subsp. *pallidum*	**-**	**-**	**?**	**+**	**-**	**+**	**+**	**+**	**-**

^a ^Combination of the presence DHFR0, DHFR1 or DHFR2; ^b ^Combination of the presence of FolC and FolC2; (+): gene identified; blankcell with (-): no gene identified; (?): predicted missing gene

### Intact folate transport and salvage

As just discussed, folate is most probably essential for all sequenced bacteria except *M. hyopneumoniae*. However, not all bacteria synthesize folate *de novo *but instead rely on an external supply [see Additional File 1, variant 001; see "Methods" for an explanation of the variant code]. To predict the absence of the *de novo *synthesis pathway, the HPPK (FolK) and DHPS (FolP) proteins were used as signature proteins (for reasons described below). Many bacteria lack homologs of both these genes (Table 1) and so almost certainly rely on reducing and glutamylating intact folates taken up from the environment. These are mainly host-associated bacteria such as *Mycoplasma *or *Treponema *or organisms that live in folate-rich environments such as *Lactobacilli*. Chloroplasts and vacuoles must likewise take up folates from the cytoplasm (Figure 4), and there is also evidence for folate uptake by intact plant cells [6].

#### (i) Transport

Systems that mediate folate uptake in auxotrophs such as *Lactobacillus casei *and *L. salivarius *have been partially biochemically characterized [33,34], but the corresponding genes remain unknown. Whatever they are, they are most likely unrelated to mammalian folate carriers (i.e., the reduced folate carrier, the folate receptor, the intestinal folate carrier, and the mitochondrial folate carrier) since these lack close homologs among bacteria and plants. However, cyanobacteria, which are folate prototrophs, have a protein with significant similarity to a folate carrier from *Leishmania *species (FT1), and the cyanobacterial protein has a close homolog in plants (52% amino acid identity), as well as several more distant relatives in plants and in alpha-, beta-, and gamma-proteobacteria. We showed first that the cyanobacterial protein (*Synechocystis slr0642*) conferred the ability to transport folates and folate analogs when expressed in *E. coli*, and then that its plant homolog (Arabidopsis *At2g32040*) did the same [35]. We further showed that the Arabidopsis At2g32040 protein is located in the chloroplast envelope [35]. The weak slr0642 homolog in some alpha-proteobacteria (*Silicibacter*, *Roseobacter*) clusters with the folate-dependent enzyme sarcosine dehydrogenase, suggesting that this protein may also be a folate transporter.

Thus, despite progress in identifying folate transporters in cyanobacteria and in the chloroplast envelope, there are as yet no candidates for the folate carriers in many folate-requiring bacterial taxa, or in plant mitochondrial, vacuolar, and plasma membranes. These still-missing genes are future prospects for discovery by comparative genomics methods [36].

#### (ii) Reduction

As noted above, DHFR is essential in both *de novo *and salvage pathways. Most bacteria have a *folA *gene (DHFR0), but two other bacterial enzymes able to reduce DHF are now known: FolM (DHFR1) belonging to the short-chain dehydrogenase/reductase (SDR) family [37], and a flavin-dependent dihydropteroate reductase that is fused to dihydropteroate synthase (DHFR2). [38]. The trypanosomatid enzyme PTR1 can also reduce DHF and folic acid [17]. As *folM *occurs in *E. coli *and other bacteria that also have a *folA *gene, its normal function is most probably not folate reduction, as discussed in a later section. The annotation of DHFR0 family members is complicated by their similarity to pyrimidine dehydrogenase family members (Pfam01872), which are numerous in Actinomycetes like *Streptomyces coelicolor*. At this stage we named them all DHFR0 but further genetic or biochemical analysis is needed to check these assignments.

Analysis of the distribution of DHFR genes in bacterial genomes reinforced the conclusions [32] that many bacteria such as *Prochlorococcus marinus *lack any recognizable DHFR proteins, and that most of these organisms use ThyX and not ThyA. Even if a high capacity for DHF reduction is not needed in ThyX-dependent organisms [39], these do require some DHFR activity to complete the *de novo *or salvage pathways so the corresponding gene(s) have yet to be identified in these organisms [32] (see Additional File 1, variants 106, 116, 006).

#### (iii) Glutamylation

FolC-like proteins can have FPGS activity alone [20] or both DHFS and FPGS activities [40], which complicates annotation. Although the bifunctional type has a unique dihydropteroate binding site [41], it overlaps the rest of the substrate binding site and we could not derive a motif to distinguish mono- and bifunctional enzymes. We therefore annotated them all as bifunctional. By analogy with the *Lactococcus. lactis *situation, we predict that organisms reliant on the salvage pathway (see Additional File 1, variants 001 and 011) will have a monofunctional FPGS. The *folC *gene is missing in the *Mycoplasma *species that contain an *fmt*, a *thyA *and a *folA *gene and must therefore rely on a salvage pathway (Table 1). This absence points to three possibilities for these species: (a) they import folate polyglutamates; (b) they have a novel type of FPGS gene; or (c) they import monoglutamyl folates and polyglutamylation is not needed. We favor the last hypothesis as there is evidence for monoglutamyl folate uptake in *Mycoplasma mycoides *[42]. A similar situation must exist in bacteria such as *Borrelia burgdorferi *that lack all folate synthesis genes but contain THF-dependent enzymes such as Fmt (Table 1).

### *De novo *folate biosynthesis

The majority of sequenced bacteria (250 out of 400) contain all genes of the pathway and are therefore predicted to be prototrophic for folate (see examples in Table 2 and Additional File 1, variant 111). However, a substantial minority lack just one or a few genes of the pterin or pABA branches, and detailed analysis of these cases reveals several biologically significant points.

**Table 2:** Examples of bacteria capable of *de novo *folate synthesis and of genes that are still missing

	***De novo early steps***						***De novo signature enzymes***		***De novo late steps***					***PABA synthesis***
**Organism**	**GCYH-I**		**DHNTPase**		**DHNA**		**DHPS**	**HPPK**	**DHFS**		**DHFR**			**PabAabc**

	**FolE**	**FolE2**	**FolQ**	FolQ2*	**FolB**	FolB2*	**FolP**	**FolK**	**FolC**	**FolC2**	**FolA**	**FolM**	**Dhfr2**	
	
*Escherichia coli K12*	**+**	**-**	**?**	**?**	**+**	**+**	**+**	**+**	**+**	**-**	**+**	**+**	**-**	**+**
*Staphylococcus aureus*	**-**	**+**	**?**	**?**	**+**	**+**	**+**	**+**	**+**	**-**	**+**	**-**	**-**	**+**
*Chlamydia trachomatis*	**?**	**?**	**?**	**?**	**+**	**+**	**+**	**+**	**-**	**+**	**+**	**-**	**-**	**?**
*Lactococcus lactis*	**+**	**-**	**+**	**-**	**+**	**+**	**+**	**+**	**+**		**+**	**-**	**-**	**?**
*Parachlamydia sp*. UWE25	**+**		**+**^#^	**-**	**+**^#^	**+**	**+**	**+**	**+**	**-**	**-**	**+**	**-**	**+**
*Helicobacter pylori*	**+**	**-**	**?**	**?**	**+**	**+**	**+^#^**	**+**	**+**	**-**	**-**	**-**	**+^#^**	**+**
*Prochlorococcus marinus*	**+**	**-**	**?**	**?**	**+**	**+**	**+**	**+**	**+**	**-**	**?**	**?**	**?**	**+**
*Clostridium perfringens*	**+**	**-**	**-**	**+**	**+**	**+**	**+**	**+**	**+**	**-**	**+**	**-**	**-**	**+**
*Rickettsia felis*	**+**	**-**	**?**	**?**	**?**	**?**	**+**	**+**	**+**	**-**	**+**	**-**	**-**	**?**
*Geobacter metallireducens*	**-**	**+**	**?**	**?**	**-**	**+**	**+**	**+**	**+**	**-**	**+**	**-**	**-**	**+**
*Mycobacterium leprae*	**+**	**-**	**?**	**?**	**+**	**+**	**FolP1**	**+**	**+**	**-**	**+**	**-**	**-**	**+**
							FolP2							
*Shewanella denitrificans*	**+**	**-**	**?**	**?**	**+**	**+**	**+**	**FolK1**	**+**		**+**	**+**	**-**	**+**
								FolK2						
*Xylella fastidiosa*	**+**	**+**	**?**	**?**	**+**	**-**	**+**	**FolK1**	**+**	**-**	**+**	**+**	**-**	**+**
								FolK2						

* Prediction not experimentally verified; ^# ^Fused proteins; Underlined and bold: physical clustering; (+): gene identified; (-): no identified gene; (?): predicted missing gene.

#### (i) The pterin branch

**Figure 5:**
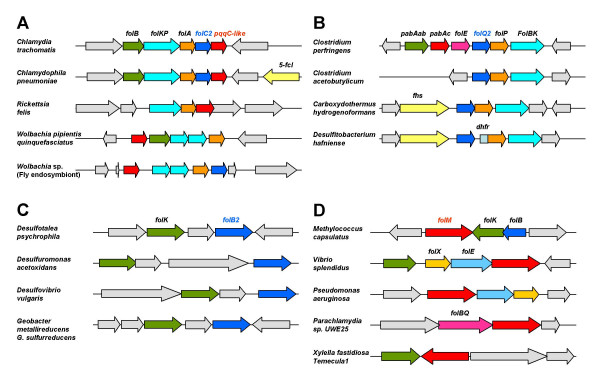
**Clustering of predicted folate-related genes with known folate synthesis genes**. Gene names are as described in the text or given below. [For full gene and genome names, see Additional File 1.] Matching colors correspond to orthologous genes. Pale grey arrows are non-folate related genes. **A**. Clustering of *folC2 *and *pqqC-like *genes. *5-fcl*, 5-formyl-THF cycloligase. **B**. Clustering of *folQ2 *genes. *fhs*, formate-tetrahydrofolate ligase; *dhfr*, dihydrofolate reductase. **C**. Clustering of *folB2 *(fructose-6-phosphate aldolase-like) genes. **D**. Clustering of *folM *genes.

The first enzyme of this branch, GCHY-I, is encoded in *E. coli *by the *folE *gene. A recent analysis of the distribution of *folE *genes among bacterial genomes showed the *folE *gene to be locally missing in one-third of them [43]. Another protein family, COG1469, was found to responsible for 7,8-dihydroneopterin triphosphate formation in these organisms. This protein was named GCHY-IB and the corresponding gene *folE2 *[43] (Table 2). Further analysis revealed that a few bacteria such as *Wolbachia*, *Chlamydia*, and *Chlamydophila *species lack both *folE *and *folE2 *homologs whereas they contain the signature genes of the pathway *folKP *(see Table 2 and additional File 1, variants 701, 702), suggesting that another family of GCHY-I enzymes has yet to be identified. For instance, at least certain *Chlamydia *species are known to synthesize folates *de novo *[44], but lack *folE *and *folE2*. A candidate for the missing GCYH-I enzyme was the *Chlamydia trachomatis *protein CT610 and its homologs, which cluster with the *folABKP *folate genes in *Chlamydia *and *Wolbachia *species (Figure 5A). The protein is homologous to the pyrroloquinoline quinone (PQQ) biosynthesis protein PqqC that catalyzes an overall eight-electron oxidation, leading to a pyrrole and pyridine ring, but their active sites are not conserved, consistent with a different enzymatic activity [45]. The CT610 gene was cloned in pBAD24 but failed to complement the dT auxotrophy of the *E. coli folE *mutant. The strong linkage of CT610 homologs with folate genes certainly points to a function in folate metabolism as other *de novo *folate genes than *folE *are missing in chlamydiae *s*uch as *folQ *or *pabAabc *(Table 2), but further studies are needed to determine its functional role.

The second step of folate synthesis is the removal of pyrophosphate. Although an enzyme mediating this step had been demonstrated in *E. coli *[46], no gene was known from any organism. We identified a DHNTP pyrophosphatase (FolQ) candidate in *L. lactis *as part of the *folKEPQC *gene cluster (Table 2) [12]. FolQ belongs to the Nudix (Nucleoside diphosphate X) hydrolase family [47]. Biochemical and genetic tests confirmed DHNTP pyrophosphatase activity [12]. Furthermore, the closest Arabidopsis homolog of *L. lactis *FolQ was also shown to have this activity [12].

Since the Nudix family is large and functionally heterogeneous it is not very amenable to projection of annotations just by homology. FolQ homologs with a high homology score occur in rather few bacteria, so that the DHNTP pyrophosphatase gene is still missing in most genomes, including *E. coli*. Other putative phosphohydrolases unrelated to FolQ, FolQ2 members of the HDIG superfamily, are found in some folate-related gene clusters (Figure 5B), such as CPE1020 in *Clostridium perfringens; *these genes are good candidates for alternatives to FolQ but again have a limited phylogenetic distribution leaving the problem open in most bacterial species (Table 2).

The third specific enzyme of the pathway, DHNA, is encoded in *E. coli *by the *folB *gene. This gene and its paralog *fol*X [13] appear to be missing in many phylogenetically diverse bacteria such as *Geobacter metallireducens*. Genome and functional context analysis allows the prediction that the DHNA role is played by members of the transaldolase (EC 2.2.1.2) family (e.g. DVU1658 in *Desulfovibrio vulgaris*). Specifically, about half the bacteria that lack DHNA have a transaldolase encoding gene that clusters with *folK *genes in several organisms (Figure 5C). This prediction awaits experimental validation as this transaldolase family is broad and only some of its members might encode a DHNA aldolase. Some genomes such as *Rickettsia felis *lack both FolB and transaldolase homologs while containing all the other *de novo *enzymes (see Table 2 and additional File 1, variant 401), again suggesting that another family of FolB enzymes has yet to be identified unless the pathway is on its way to elimination in these organisms specifically.

HPPK (FolK) and DHPS (FolP) are distinctive proteins found in all organisms that make folate *de novo *and so, as noted above, these were used as pathway signature genes. A few sporadic organisms apparently lack one of the two genes, but further analysis shows that this is usually because of a gene-calling problem (a homolog can be found using the tblastn algorithm) or because the corresponding genome is still incomplete. Some organisms, however, have two *folP *genes or two *folK *genes (Table 2). Are these functionally redundant or catalyzing different reactions? In most cases one paralog is clustered with folate genes and the other clusters with genes involved in different pathways (see Table 2 and additional File 1). For instance, in the high-GC gram-positive group the second *folP *(*folP2*) clusters with cell wall synthesis genes. In *Mycobacterium leprae *the *folP2 *gene does not complement an *E. coli folP *mutant whereas the copy that clusters with the folate genes (*folP1*) does, suggesting that *folP2 *is involved in another pathway [49].

FolK is duplicated in many organisms. In most cases such as *Shewanella denitrificans *(Table 2), one copy is in a folate operon and the other in a pantothenate operon but there are several cases where both genes are close to other folate biosynthesis genes (see also Additional File 1). Only experimental testing will show whether both copies are active. It is of note that an internal duplication of FolK and fusion with FolB is found in *Bifidobacterium longum*.

**Figure 6:**
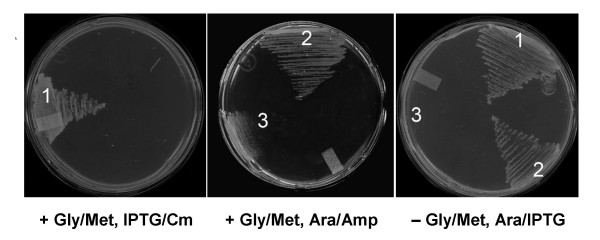
**Complementation of *folC *function by *Chlamydia trachomatis CT611***. Complementation of *E. coli folC *mutant SF4 by a pBAD24 plasmid harboring *CT611 *from *Chlamydia trachomatis *on MS minimal medium with or without glycine plus methionine. *E. coli folC *was included as a positive control. 1, pCA24N::*EcfolC*; 2, pBAD24::*CT611*; 3, pBAD24 alone. SF4 shows slower growth even in the presence of the added amino acids. The appropriate antibiotics and inducers were included in the media as indicated. Amp, ampicillin; Ara, arabinose; Cm, chloramphenicol.

The sequenced chlamydiae all lack homologs of *folC *(DHFS/FPGS) but have *folPK *homologs (see Table 2 and additional File 1), making *folC *a locally missing gene in this group. Inspection revealed that a member of gene family COG1478 is clustered in chlamydiae with folate biosynthesis genes (Figure 5A, *folC2*). This COG1478 family contains the F_420_:γ-glutamyl ligase CofE of Archaea and *Mycobacteria *[50]. CofE catalyzes the GTP-dependent successive addition of two γ-linked L-glutamates to the L-lactyl phosphodiester of 7,8-didemethyl-8-hydroxy-5-deazariboflavin (F_420_), a reaction analogous to that mediated by FolC. Chlamydiae almost certainly do not make F_420 _since they lack all the other known *cof *genes [50]. We accordingly predicted that the CofE homolog in chlamydiae has FolC activity. A *cofE *homolog (CT611) was shown to complement the methionine and glycine requirements of the *E. coli folC *mutant SF4 [40] indicating that CT611 can indeed functionally replace FolC (Figure 6). The *E. coli folC *gene from the ASKA collection [51] was used as a positive control.

#### (ii) The pABA branch

**Figure 7:**
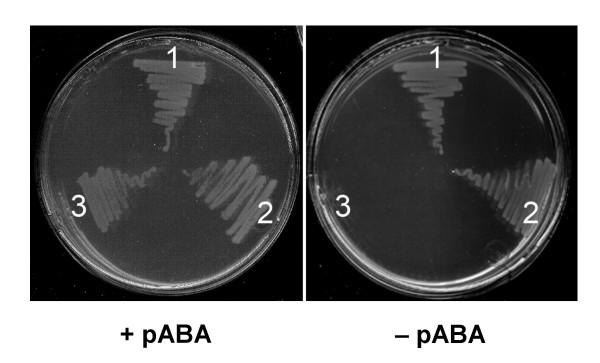
**Complementation of *pabAa pabAb *function by *Corynebacterium diphtheriae DIP1790***. Complementation of *E. coli pabAa pabAb *double mutant BN1163 by a pLOI707HE plasmid harboring *DIP1790 *from *Corynebacterium diphtheriae *on M9 minimal medium with or without pABA. Arabidopsis *ADCS *was included as a positive control. 1, pLOI707HE::*ADCS*; 2, pLOI707HE::*DIP1790*; 3, pLOI707HE alone. The medium contained IPTG and appropriate antibiotics.

We adopted the nomenclature of Xie *et al*. [52] for the pABA branch genes. These genes are hard to annotate for several reasons. In the first place, they can be fused in various combinations. A fusion between the subunits of ADC synthase (PabAa and PabAb) is a common arrangement, as is fusion between PabAa and ADC lyase (PabAc). In one genome, *Corynebacterium diphtheriae*, our analysis indicated a triple fusion. The functions of this PabAa-PabAb-PabAc fusion gene (DIP1790) were tested experimentally. The gene was cloned into an expression vector and introduced into an *E. coli pabAa pabAb *mutant (strain BN1163), which cannot grow on minimal medium unless it expresses a recombinant enzyme with ADC synthase activity. A bifunctional PabAa-PabAb ADC synthase protein from Arabidopsis served as a positive control. Like the positive control, expression of the DIP1790 protein restored pABA prototrophy (Figure 7). This result shows that the DIP1790 protein has ADC synthase activity but does not demonstrate ADC lyase activity because the BN1163 strain has endogenous ADC lyase (PabAc). Enzyme assays were therefore used to test DIP1790 for ADC lyase activity. BN1163 cultures harboring plasmids encoding DIP1790, Arabidopsis ADC synthase, and *E. coli *PabAc were grown and induced, and proteins were extracted. Extracts of cells expressing DIP1790 were incubated with chorismate and glutamine, without or with *E. coli *PabAc; pABA was formed in the absence of PabAc whereas, as expected, Arabidopsis PabAa-PabAb formed pABA only if PabAc was added. Reaction rates (nmol pABA h^-1 ^mg^-1 ^protein) were: DIP1790 – PabAc, 7.0; DIP170 + PabAc, 6.0; Arabidopsis ADCS – PabAc, <0.01; Arabidopsis ADCS + PabAc, 4.0. These data establish that DIP1790 has ADC lyase as well as ADC synthase activity.

Another difficulty in annotating the *pabAabc *genes is that most organisms contain paralogs of p*abAa *and *pabAb *(*trpAa *and *trpAb*, respectively) that participate in tryptophan biosynthesis [52], and in some cases the PabAb (amidotransferase) subunit is shared between the pABA and tryptophan pathways [53]. Finally, PabAc belongs to the large branched-chain amino acid aminotransferase family (EC 2.6.1.42) and is hard to distinguish from these enzymes. These problems mean that the current SEED annotation of the pABA branch of folate synthesis should be taken as tentative. That said, analysis of the distribution of these genes reveals that most bacteria make pABA from chorismate. As expected, many intracellular bacteria lack all *pabA *genes. In cases where the organisms have the pterin branch but lack all enzymes of the pABA branch, annotation problems cannot be ruled out but an alternative pathway for the biosynthesis of pABA, starting for example with dehydroquinate instead of chorismate, could also be the answer [54].

### Pterin salvage

**Figure 8:**
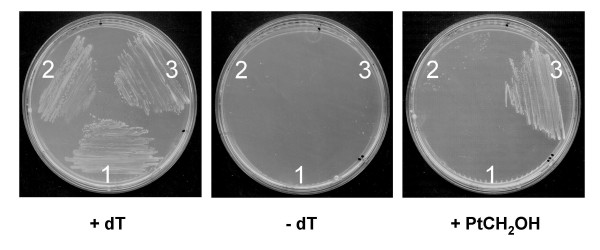
**Pterin utilization by an *E. coli folE *deletant harboring *Xylella fastidiosa PD0677 *or *Leishmania major PTR1***. Deletant cells transformed with pBluescript (pBS) alone, or pBS harboring *Xylella fastidiosa *Temecula1 *PD0677 *or *Leishmania major PTR1*, were streaked on LB medium containing IPTG and appropriate antibiotics, without or with 300 μM thymidine (dT) or 19 μM 6-hydroxymethylpterin (HMPt). For *PD0677*, neopterin, monapterin, and pterin-6-aldehyde were also tested and found not to support growth (not shown). 1, pBS::*PD0677*; 2, pBS; 3, pBS::*PTR1*.

The *Leishmania *pterin reductase PTR1 is a member of the SDR family, but has a highly characteristic motif TGX_3_RXG (in place of the TGX_3_GXG motif that is typical of this family) [55]. This motif is shared with *E. coli *FolM and similar SDR family proteins in a variety of bacterial taxa. Several of the *folM*-like genes are clustered with genes of the pterin branch of folate synthesis (Figure 8), suggesting a function in folate or pterin synthesis Since *E. coli *[56] and other bacteria [57] are known to contain tetrahydromonapterin or other tetrahydropterins that could serve as cofactors for pterin-dependent enzymes, we predict that *folM*-like genes are not primarily involved in folate synthesis but rather are pteridine reductases that, like PTR1, produce and/or reduce 7,8-dihydropterins. (Note that such reductases are distinct from 6,7-dihydropterin reductases [also termed quinonoid pteridine reductases], of which *E. coli *has two [58,59].)

Consistent with this prediction, the recombinant FolM protein catalyzes reduction of dihydrobiopterin to the tetrahydro form; unlike PTR1, however, it does not mediate reduction of fully oxidized biopterin to the dihydro form [37]. Supporting the latter observation, we found that an *E. coli *GCHY-I mutant (which is unable to make pterins) can use the dihydro but not the oxidized forms of neopterin, monapterin, or 6-hydroxymethylpterin to support folate synthesis [60]. Futhermore, expression of a typical *folM*-like gene (*Xylella fastidiosa *PD0677, Figure 5D, Table 2) from a plasmid did not enable this mutant to use oxidized pterins, indicating that – like FolM – the PD0677 gene product does not act on oxidized pterins (Figure 8). In control experiments in which *Leishmania *PTR1 was expressed from a plasmid, the mutant was able to use oxidized pterins, confirming that it is oxidized pterin reduction (and not uptake) that is lacking in *E. coli *(Figure 8) [60].

Searching the Arabidopsis genome revealed some 86 members of the SDR family, of which none had the TGX_3_RXG motif. This led to the prediction that Arabidopsis would be unable to salvage oxidized pterins, which was verified by showing that 6-hydroxymethylpterin was not reduced *in vivo *or *in vitro*, and was not incorporated into folates [60].

## Conclusion

This analysis and integration study demonstrates that simple phylogenomic analysis of a biochemical pathway – even a well-known one – can unearth globally missing (e.g., *folQ*) or locally missing (e.g., *folE2 or folC2*) genes in bacteria and plants and reveals that many open questions remain (such as the missing *folQ*, *folB*, *folE *cases listed in Table 2). It can also identify, or suggest functions for, additional genes related to the pathway (e.g., *folM*). Such analysis can thus lead to discovery of potential new drug or herbicide targets such as GCHY-IB, which occurs in many pathogenic bacteria but not in mammals, or the chloroplast folate carrier that is likewise absent from mammals.

It should be noted that content of the current SEED folate subsystem captures the present status of an ongoing annotation effort, that the content will be refined and improved as more bacterial and plant genomes are added, and that further predictions are expected to emerge. Finally, we emphasize that the predictions herein are offered with the hope that others will find them useful in their own research.

## Methods

### Bioinformatics

Analysis of the folate subsystem was performed in the SEED database [61]. Results are available in the 'Folate biosynthesis sub-system' on the public SEED server at [62]. The snapshot of this analysis on the SEED database is given in the additional file. Phylogenetic pattern searches were made on the NMPDR SEED server at [63] to find candidates for the missing *folE *and *folC *genes. We also used the Blast tools and resources at NCBI [64] and the comparative genomics platforms STRING [65] for additional gene clustering analysis tools.

Annotations for paralog families were made using physical clustering on the chromosome when possible or by building phylogenetic trees using the ClustalW tool [66] integrated in SEED or deriving specific protein motifs. Pseudogenes (i.e., those encoding clearly aberrant proteins) were ignored; these are not uncommon in the folate pathways of intracellular parasites undergoing genome reduction [67].

The 'variant code' is used in SEED to schematize the type of pathways found in a given organism [21]. A three-digit code was used. Digit one describes the pterin branch of the pathway: 1 = complete, 0 = HPPK and DHPS missing, 4 = DHNA missing, 7 = GCHY-I missing. Digit two describes the pABA branch: 1 = two or three of the *pabAabc *genes present, 0 = all *pabAabc *genes missing or just one present. Digit three describes the salvage pathway: 1 = complete; 0 = FPGS and DHFR missing; 2 = FPGS missing; 6 = DHFR missing. Variant -1 represents genomes with no pathway genes but no need for them because no folate-dependent enzymes are present. Particular care was given to annotation of fused proteins, which are common in both branches of the pathway; SEED has annotation tools to deal with fusion proteins [21].

### Strains, growth conditions, and cloning

Bacteria were routinely grown at 37°C in LB medium (BD Diagnostic Systems), in minimal medium [68] supplemented with 0.2% (v/v) glycerol, or in M9 medium [35]. Agar (BD Diagnostic Systems) concentration in plates was 15 g l^-1^. Transformations were by standard procedures [69,70]. Thymidine (dT, 300 μM), ampicillin (100 μg ml^-1^), kanamycin (50 or 100 μg ml^-1^), tetracycline (10 μg ml^-1^), isopropyl-β-D-thiogalactopyranoside (IPTG, 0.5 or 1 mM), methionine (100 μg ml^-1^), glycine (100 μg ml^-1^), pABA (0.5 μg ml^-1^) and L-arabinose (0.02–0.2%, w/v) were added as required. Strains Topo10 (Invitrogen), BL21-CodonPlus (DE3)-RIL (Stratagene), DH10B, or DH5α were used for cloning and expression. SF4 (F^-^*strA recA folC srlC*::Tn10) [40], BN1163 (*pabA1*, *pab*^-^*B::Kan*, *rpsL704*, *ilvG-*, *rfb-50*, *rph-1*) (B. Nichols, University of Chicago), and MG1655 (*ΔfolE*::Kan^R^) [35] were used for complementation tests.

The *Chlamydia trachomatis *CT610 and CT611 genes were cloned in pBAD24 [71] using the following primers: CT610, 5'-AATA*CCATGG*TGGAGGTGTTTATGAA-3' and 5'-AATA*AAGCTT*TTAATAAGATTGATGACAACTAC-3'; CT611, 5'-AATA*CCATGG*AAATAACTCCGATCAAAACAC-3' and 5'-AATA*AAGCTT*TCATTTCTTTTCTTGACTCCAC-3'. Genomic DNA from *C. trachomatis*, LGV-II, strain 434 was obtained from ABI (Maryland). PCR products were obtained and purified as described [43], then digested with *Nco*I/*Hin*dIII before ligation into plasmid pBAD24 digested with the same enzymes and transformation into Topo10 cells (Invitrogen). The respective plasmids named pBY149.9 (expressing CT610) and pBY143.1 (expressing CT611) were checked by sequencing.

The *Corynebacterium diphtheriae *DIP1790 gene was cloned into pGEM-T Easy (Promega), after amplification from genomic DNA (obtained from the American Type Culture Collection) using the primers 5'-*GCGGCCGC*CACAGGAAACAGCTATGGTTATGCAACGCGCGCA-3' and 5'-*GAGCTC*TCACACTTGGGCGATATTCT-3'. The *Sst*I site in the gene was ablated by PCR using the internal primers 5'-TCATCACCGAaCtTGAAGGCA-3' and 5'-TTTGCCTTCAaGtTCGGTGATG-3' (changed nucleotides in lower case). The modified gene was ligated into pGEM-T Easy and verified by sequencing. It was then excised with *Not*I and *Sst*I and ligated into pLOI707HE [72]. This construct was used to transform *E. coli *BN1163. Complementation tests were made using minimal medium, appropriately supplemented as above.

The *Xylella fastidiosa *Temecula1 PD0677 amplicon preceded by a Shine-Dalgarno sequence and a stop codon in frame with LacZα was cloned between the *Eco*RI and *Kpn*I sites of pBluescript SK-. The PCR template was genomic DNA from the American Type Culture Collection; primers were 5'-AGTCA*GAATTC*GTGAAGGAAACAGCTATGTCAGATCCCTCTAAAGTC-3' and 5'-AGTA*GGTACC*TCATGTCAGCGTGCGGCC-3'; amplification was with KOD HiFi polymerase. The deduced amino acid sequence differed from that published in having serine not isoleucine at position 57. The PTR1 construct was as described [60]. The constructs were introduced into *E. coli folE *deletant cells [35]. Transformants were grown on LB plates supplemented appropriately as above.

### ADCS/ADCL enzyme assays

Protein extracts were prepared from IPTG-induced cultures as described [73] from strain BN1163 harboring three pLOI707HE constructs – DIP1790, Arabidopsis ADCS [73], or vector alone – and from BL21-CodonPlus (DE3)-RIL harboring *E. coli *PabC cloned in pJMG30 [74]. Glutamine-dependent pABA synthesis activity was assayed as described [73]. PabC extract (3.8 μg protein) was added when indicated. Assays were incubated for 1 h at 37°C, stopped with 20 μl of 75% (v/v) acetic acid, held on ice for 1 h, then stored at -80°C until analysis. pABA was estimated by HPLC with fluorescence detection [73].

## Authors' contributions

BElY carried out the complementation studies on the *Chlamydia cofE *homolog. RDdelaG made the complementation and biochemical assays on the *Corynebacterium *pABA synthesis protein. AN carried out the studies on the *Xylella folM *homolog. VdeC-L and ADH conceived the study, carried out bioinformatic work, and drafted the manuscript. All authors read and approved the final manuscript.

## Supplementary Material

Additional File 1 Spreadsheet summarizing the distribution, clustering, and fusions of bacterial and plant folate synthesis genes. This table represents a snapshot for the record of the "BMCgenomics 2007" table that can be found in the Folate Biosynthesis sub-system on the public SEED website . Clustering is shown by similar color backgrounds. Genome and protein IDs are from the SEED database. Abbreviations for the functional roles are given in the first page of the spreadsheet, gene distribution in all analyzed genomes in the second page. Note that the SEED table is the primary source to which the reader is directed; it is not static but develops with time as new genomes become available and predictions are tested and validated. Click here for file

## References

[B1] Matthews RG, Neidhardt FC, Curtiss R 3rd, Ingraham JL, Lin ECC, Low KB, Magasanik B, Reznikoff WS, Riley M, Schaechter M, Umbarger HE (1996). One-carbon metabolism. Escherichia coli and Salmonella: Cellular and Molecular Biology.

[B2] Huennekens FM, Vitols KS, Pope LE, Fan J (1992). Membrane transport of folate compounds. J Nutr Sci Vitaminol (Tokyo).

[B3] Lucock M (2000). Folic acid: nutritional biochemistry, molecular biology, and role in disease processes. Mol Genet Metab.

[B4] Cossins EA, Chen L (1997). Folates and one-carbon metabolism in plants and fungi. Phytochemistry.

[B5] Green JC, Nichols BP, Matthews RG, Neidhardt FC, Curtiss R 3rd, Ingraham JL, Lin ECC, Low KB, Magasanik B, Reznikoff WS, Riley M, Schaechter M, Umbarger HE (1996). Folate biosynthesis, reduction, and polyglutamylation. Escherichia coli and Salmonella: Cellular and Molecular Biology.

[B6] Hanson AD, Gregory JF (2002). Synthesis and turnover of folates in plants. Curr Opin Plant Biol.

[B7] Hyde JE (2005). Exploring the folate pathway in *Plasmodium falciparum*. Acta Trop.

[B8] Huovinen P, Sundström L, Swedberg G, Sköld O (1995). Trimethoprim and sulfonamide resistance. Antimicrob Agents Chemother.

[B9] Sybesma W, Starrenburg M, Kleerebezem M, Mierau I, de Vos WM, Hugenholtz J (2003). Increased production of folate by metabolic engineering of *Lactococcus lactis*. Appl Environ Microbiol.

[B10] Hossain T, Rosenberg I, Selhub J, Kishore G, Beachy R, Schubert K (2004). Enhancement of folates in plants through metabolic engineering. Proc Natl Acad Sci USA.

[B11] Díaz de la Garza R, Quinlivan EP, Klaus SM, Basset GJ, Gregory JF, Hanson AD Folate biofortification in tomatoes by engineering the pteridine branch of folate synthesis. Proc Natl Acad Sci USA.

[B12] Klaus SM, Wegkamp A, Sybesma W, Hugenholtz J, Gregory JF, Hanson AD (2005). A nudix enzyme removes pyrophosphate from dihydroneopterin triphosphate in the folate synthesis pathway of bacteria and plants. J Biol Chem.

[B13] Haussmann C, Rohdich F, Schmidt E, Bacher A, Richter G (1998). Biosynthesis of pteridines in *Escherichia coli*. Structural and mechanistic similarity of dihydroneopterin-triphosphate epimerase and dihydroneopterin aldolase. J Biol Chem.

[B14] Ferone R, Singer SC, Hunt DF (1986). *In vitro *synthesis of alpha-carboxyl-linked folylpolyglutamates by an enzyme preparation from *Escherichia coli*. J Biol Chem.

[B15] Ravanel S, Cherest H, Jabrin S, Grunwald D, Surdin-Kerjan Y, Douce R, Rebeille F (2001). Tetrahydrofolate biosynthesis in plants: molecular and functional characterization of dihydrofolate synthetase and three isoforms of folylpolyglutamate synthetase in *Arabidopsis thaliana*. Proc Natl Acad Sci USA.

[B16] Orsomando G, de la Garza RD, Green BJ, Peng M, Rea PA, Ryan TJ, Gregory JF, Hanson AD (2005). Plant gamma-glutamyl hydrolases and folate polyglutamates: characterization, compartmentation, and co-occurrence in vacuoles. J Biol Chem.

[B17] Nare B, Hardy LW, Beverley SM (1997). The roles of pteridine reductase 1 and dihydrofolate reductase-thymidylate synthase in pteridine metabolism in the protozoan parasite *Leishmania major*. J Biol Chem.

[B18] Orsomando G, Bozzo GG, de la Garza RD, Basset GJ, Quinlivan EP, Naponelli V, Rébeillé F, Ravanel S, Gregory JF, Hanson AD (2006). Evidence for folate-salvage reactions in plants. Plant J.

[B19] Green JM, Ballou DP, Matthews RG (1988). Examination of the role of methylenetetrahydrofolate reductase in incorporation of methyltetrahydrofolate into cellular metabolism. FASEB J.

[B20] Toy J, Bognar AL (1990). Cloning and expression of the gene encoding *Lactobacillus casei *folylpoly-gamma-glutamate synthetase in *Escherichia coli *and determination of its primary structure. J Biol Chem.

[B21] Overbeek R, Begley T, Butler RM, Choudhuri JV, Chuang HY, Cohoon M, de Crécy-Lagard V, Diaz N, Disz T, Edwards R (2005). The subsystems approach to genome annotation and its use in the project to annotate 1000 genomes. Nucleic Acids Res.

[B22] Galperin MY, Brenner SE (1998). Using metabolic pathway databases for functional annotation. Trends Genet.

[B23] Selkov E, Maltsev N, Olsen GJ, Overbeek R, Whitman WB (1997). A reconstruction of the metabolism of *Methanococcus jannaschii *from sequence data. Gene.

[B24] Gerlt JA, Babbitt PC (2000). Can sequence determine function?. Genome Biol.

[B25] Karp PD (2004). Call for an enzyme genomics initiative. Genome Biol.

[B26] Osterman A, Overbeek R (2003). Missing genes in metabolic pathways: a comparative genomics approach. Curr Opin Chem Biol.

[B27] Koonin EV, Mushegian AR, Bork P (1996). Non-orthologous gene displacement. Trends Genet.

[B28] Clark BF, Marcker KA (1966). The role of N-formyl-methionyl-sRNA in protein biosynthesis. J Mol Biol.

[B29] Vasconcelos AT, Ferreira HB, Bizarro CV, Boniato SL, Carvalho MO, Pinto PM, Almeida DF, Almeida LG, Almeida R, Alves-Filho L (2005). Swine and poultry pathogens: the complete genome sequences of two strains of *Mycoplasma hyopneumoniae *and a strain of *Mycoplasma synoviae*. J Bacteriol.

[B30] Schirch L, Gross T (1968). Serine transhydroxymethylase. Identification as the threonine and allothreonine aldolases. J Biol Chem.

[B31] Myllykallio H, Lipowski G, Leduc D, Filee J, Forterre P, Liebl U (2002). An alternative flavin-dependent mechanism for thymidylate synthesis. Science.

[B32] Myllykallio H, Leduc D, Filee J, Liebl U (2003). Life without dihydrofolate reductase FolA. Trends Microbiol.

[B33] Henderson GB, Zevely EM, Huennekens FM (1977). Purification and properties of a membrane-associated, folate-binding protein from *Lactobacillus casei*. J Biol Chem.

[B34] Kumar HP, Tsuji JM, Henderson GB (1987). Folate transport in *Lactobacillus salivarius*. Characterization of the transport mechanism and purification and properties of the binding component. J Biol Chem.

[B35] Klaus SM, Kunji ER, Bozzo GG, Noiriel A, de la Garza RD, Basset GJ, Ravanel S, Rébeillé F, Gregory JF, Hanson AD (2005). Higher plant plastids and cyanobacteria have folate carriers related to those of trypanosomatids. J Biol Chem.

[B36] Rodionov DA, Hebbeln P, Gelfand MS, Eitinger T (2006). Comparative and functional genomic analysis of prokaryotic nickel and cobalt uptake transporters: evidence for a novel group of ATP-binding cassette transporters. J Bacteriol.

[B37] Giladi M, Altman-Price N, Levin I, Levy L, Mevarech M (2003). FolM, a new chromosomally encoded dihydrofolate reductase in *Escherichia coli*. J Bacteriol.

[B38] Levin I, Mevarech M, Palfey BA (2007). Characterization of a novel bifunctional dihydropteroate synthase/dihydropteroate reductase enzyme from *Helicobacter pylori*. J Bacteriol.

[B39] Graziani S, Xia Y, Gurnon JR, Van Etten JL, Leduc D, Skouloubris S, Myllykallio H, Liebl U (2004). Functional analysis of FAD-dependent thymidylate synthase ThyX from *Paramecium bursaria *chlorella virus-1. J Biol Chem.

[B40] Bognar AL, Osborne C, Shane B, Singer SC, Ferone R (1985). Folylpoly-gamma-glutamate synthetase-dihydrofolate synthetase. Cloning and high expression of the *Escherichia coli folC *gene and purification and properties of the gene product. J Biol Chem.

[B41] Mathieu M, Debousker G, Vincent S, Viviani F, Bamas-Jacques N, Mikol V (2005). *Escherichia coli FolC *structure reveals an unexpected dihydrofolate binding site providing an attractive target for anti-microbial therapy. J Biol Chem.

[B42] Neale GA, Mitchell A, Finch LR (1981). Formylation of methionyl-transfer ribonucleic acid in *Mycoplasma mycoides *subsp. *mycoides*. J Bacteriol.

[B43] El Yacoubi B, Bonnett S, Anderson JN, Swairjo MA, Iwata-Reuyl D, de Crécy-Lagard V (2006). Discovery of a new prokaryotic type I GTP cyclohydrolase family. J Biol Chem.

[B44] Fan H, Brunham RC, McClarty G (1992). Acquisition and synthesis of folates by obligate intracellular bacteria of the genus *Chlamydia*. J Clin Invest.

[B45] Schwarzenbacher R, Stenner-Liewen F, Liewen H, Robinson H, Yuan H, Bossy-Wetzel E, Reed JC, Liddington RC (2004). Structure of the *Chlamydia *protein CADD reveals a redox enzyme that modulates host cell apoptosis. J Biol Chem.

[B46] Suzuki Y, Brown GM (1974). The biosynthesis of folic acid. XII. Purification and properties of dihydroneopterin triphosphate pyrophosphohydrolase. J Biol Chem.

[B47] Bessman MJ, Frick DN, O'Handley SF (1996). The MutT proteins or "Nudix" hydrolases, a family of versatile, widely distributed, "housecleaning" enzymes. J Biol Chem.

[B48] Tucker AM, Winkler HH, Driskell LO, Wood DO (2003). *S*-Adenosylmethionine transport in *Rickettsia prowazekii*. J Bacteriol.

[B49] Williams DL, Spring L, Harris E, Roche P, Gillis TP (2000). Dihydropteroate synthase of *Mycobacterium leprae *and dapsone resistance. Antimicrob Agents Chemother.

[B50] Li H, Graupner M, Xu H, White RH (2003). CofE catalyzes the addition of two glutamates to F420-0 in F420 coenzyme biosynthesis in *Methanococcus jannaschii*. Biochemistry.

[B51] Kitagawa M, Ara T, Arifuzzaman M, Ioka-Nakamichi T, Inamoto E, Toyonaga H, Mori H (2005). Complete set of ORF clones of *Escherichia coli *ASKA library (a complete set of *E. coli *K-12 ORF archive): unique resources for biological research. DNA Res.

[B52] Xie G, Keyhani NO, Bonner CA, Jensen RA (2003). Ancient origin of the tryptophan operon and the dynamics of evolutionary change. Microbiol Mol Biol Rev.

[B53] Yanofsky C (2001). Advancing our knowledge in biochemistry, genetics, and microbiology through studies on tryptophan metabolism. Annu Rev Biochem.

[B54] Porat I, Sieprawaska-Lupa M, Teng Q, Bohanon FJ, White RH, Whitman WB (2006). Biochemical and genetic characterization of an early step in a novel pathway for the biosynthesis of aromatic amino acids and p-aminobenzoic acid in the archaeon *Methanococcus maripaludis*. Mol Microbiol.

[B55] Gourley DG, Schuttelkopf AW, Leonard GA, Luba J, Hardy LW, Beverley SM, Hunter WN (2001). Pteridine reductase mechanism correlates pterin metabolism with drug resistance in trypanosomatid parasites. Nat Struct Biol.

[B56] Ikemoto K, Sugimoto T, Murata S, Tazawa M, Nomura T, Ichinose H, Nagatsu T (2002). (6*R*)-5,6,7,8-tetrahydro-L-monapterin from *Escherichia coli*, a novel natural unconjugated tetrahydropterin. Biol Chem.

[B57] Lee HW, Oh CH, Geyer A, Pfleiderer W, Park YS (1999). Characterization of a novel unconjugated pteridine glycoside, cyanopterin, in *Synechocystis *sp. PCC 6803. Biochim Biophys Acta.

[B58] Vasudevan SG, Shaw DC, Armarego WL (1988). Dihydropteridine reductase from *Escherichia coli*. Biochem J.

[B59] Vasudevan SG, Armarego WL, Shaw DC, Lilley PE, Dixon NE, Poole RK (1991). Isolation and nucleotide sequence of the *hmp *gene that encodes a haemoglobin-like protein in *Escherichia coli *K-12. Mol Gen Genet.

[B60] Noiriel A, Naponelli V, Gregory JF, Hanson AD (2007). Pterin and folate salvage: plants and *Escherichia coli *lack capacity to reduce oxidized pterins. Plant Physiol.

[B61] http://anno-3.nmpdr.org/anno/FIG/subsys.cgi.

[B62] http://theseed.uchicago.edu/FIG/index.cgi.

[B63] http://www.nmpdr.org/FIG/sigs.cgi?SPROUT=1.

[B64] Altschul SF, Madden TL, Schaffer AA, Zhang J, Zhang Z, Miller W, Lipman DJ (1997). Gapped BLAST and PSI-BLAST: a new generation of protein database search programs. Nucleic Acids Res.

[B65] von Mering C, Jensen LJ, Snel B, Hooper SD, Krupp M, Foglierini M, Jouffre N, Huynen MA, Bork P (2005). STRING: known and predicted protein-protein associations, integrated and transferred across organisms. Nucleic Acids Res.

[B66] Chenna R, Sugawara H, Koike T, Lopez R, Gibson TJ, Higgins DG, Thompson JD (2003). Multiple sequence alignment with the Clustal series of programs. Nucleic Acids Res.

[B67] Davis RE, Jomantiene R, Zhao Y (2005). Lineage-specific decay of folate biosynthesis genes suggests ongoing host adaptation in phytoplasmas. DNA Cell Biol.

[B68] Richaud C, Mengin-Lecreulx D, Pochet S, Johnson EJ, Cohen GN, Marlière P (1993). Directed evolution of biosynthetic pathways. Recruitment of cysteine thioethers for constructing the cell wall of *Escherichia coli*. J Biol Chem.

[B69] Miller JH (1972). Experiments in Molecular Genetics.

[B70] Sambrook J, Fitsch EF, Maniatis T (1989). Molecular Cloning: A Laboratory Manual.

[B71] Guzman LM, Belin D, Carson MJ, Beckwith J (1995). Tight regulation, modulation, and high-level expression by vectors containing the arabinose PBAD promoter. J Bacteriol.

[B72] Arfman N, Worrell V, Ingram LO (1992). Use of the *tac *promoter and *lacIq *for the controlled expression of *Zymomonas mobilis *fermentative genes in *Escherichia coli *and *Zymomonas mobilis*. J Bacteriol.

[B73] Basset GJ, Ravanel, Quinlivan EP, White R, Giovannoni JJ, Rébeillé F, Nichols BP, Shinozaki K, Seki M, Gregory JF, Hanson AD (2004). Folate synthesis in plants: the last step of the *p*-aminobenzoate branch is catalyzed by a plastidial aminodeoxychorismate lyase. Plant J.

[B74] Green JM, Merkel WK, Nichols BP (1992). Characterization and sequence of *Escherichia coli pabC*, the gene encoding aminodeoxychorismate lyase, a pyridoxal phosphate-containing enzyme. J Bacteriol.

